# Levels of HOXB7 and miR-337 in pancreatic ductal adenocarcinoma patients

**DOI:** 10.1186/1746-1596-9-61

**Published:** 2014-03-18

**Authors:** Rui Zhang, Shangen Zheng, Yuwen Du, Yuanyuan Wang, Wenqiao Zang, Guoqiang Zhao

**Affiliations:** 1 Department of emergency, The First Affiliated Hospital of Zhengzhou University, No.1 Jianshe Road, Zhengzhou, Henan 450052, China; 2 Department of Blood Transfusion, Wuhan General Hospital of Guangzhou Military Command, Wuhan, Hubei 430070, China; 3 College of Basic Medical Sciences, Zhengzhou University, No.100 Kexue Road, Zhengzhou 450001, China

**Keywords:** Pancreatic ductal adenocarcinoma (PDAC), HOXB7, miR-337

## Abstract

**Background:**

Many studies have revealed that homeobox-B7 (HOXB7) and miR-337 play important roles in different types of human cancers. However, the relationship of HOXB7 and miR-337 in PDAC with clinicopathological factors has not yet been examined and their biological roles remain to be explored.

**Methods:**

Using quantitative real-time RT-PCR and immunohistochemical staining, the expression of HOXB7 mRNA, miR-337, and HOXB7 protein in 44 PDAC samples was detected. Survival curves were made using follow-up data. The relationship between clinical or pathological characteristics and the prognosis was analyzed.

**Results:**

The expression levels of HOXB7 mRNA and HOXB7 protein were significantly elevated in PDAC samples than that in non-malignant adjacent tissues. There were obvious differences in HOXB7 mRNA and proteins between tumors of different diameters, differentiation, TNM stage, and lymph node status. The level of miR-337 was markedly lower in tumor samples than in non-malignant adjacent tissues. The expression of miR-337 was related to TNM stage and lymph node status. There were significant differences in survival curves between patients with tumors <4 cm in diameter and patients with tumors ≥4 cm, among groups of well, moderately, and poorly differentiated tumors, between groups with TNM stages I, II and III or IV, between groups with metastatic lymph nodes and non-metastatic lymph nodes, among groups of HOXB7 protein expression negative (or weak) and positive, between groups with low levels of miR-337 expression and with high levels of miR-337 expression. The levels of HOXB7 mRNA, HOXB7 protein, and miR-337 were found to be associated with longer survival.

**Conclusion:**

The present study showed that HOXB7 was over-expressed and miR-337 was minimally expressed in PDAC tissues, and their levels were related to TNM stage and lymph node status. The levels of HOXB7 mRNA, HOXB7 protein, and miR-337 were associated with survival in PDAC patients. Results suggested that HOXB7 and miR-337 could be used as determinants of PDAC patient prognosis.

**Virtual slides:**

The virtual slides for this article can be found here: http://www.diagnosticpathology.diagnomx.eu/vs/1509730773118658.

## Introduction

Pancreatic ductal adenocarcinoma (PDAC) is one of the most common causes of cancer-related death in the world, and only <3% of patients survive for five years after diagnosis. PDAC is usually diagnosed at an advanced stage and most patients are unsuitable for curative surgery [1,2].

Many previous studies have evaluated the expression and functions of proteins, genes, and enzymes in PDAC. The homeobox gene family has 39 members. They are classified into four groups (HOX-A, HOX-B, HOX-C, and HOX-D) located on chromosomes 7p, 17q, 12q, and 2q, respectively. The HOX genes encode transcriptional factors that regulate gene expression and control morphogenesis and cell differentiation. Aberrant expression of HOX genes has been reported in many human cancers [3-8] including hepatocellular carcinoma [9], ovarian carcinoma [10], breast cancer [11], and leukemia [12]. Studies have shown that HOXB7 plays an important role in transformation, proliferation, and survival of tumor cells [13-18]. MicroRNAs (miRNAs) are short, non-coding RNAs that regulate gene expression post-transcriptionally by binding to the 3′UTR of target mRNAs [19,20]. MiRNAs were found to play fundamentally important roles in the pathogenesis, progression and metastasis of PDAC [21-24]. Many studies have shown miR-337 to be involved in tumor cell proliferation, migration, and invasion [25]. Its expression was found to be related to the tumor prognosis in some patients [26-28]. miR-337 has been identified as a key regulator of HOX and TGFBR2 [29,30]. However, the relationship between the expression of HOXB7 mRNA and miR-337 and clinicopathological factors is still unclear. The biological roles played by HOXB7 and miR-337 in PDAC remian to be examined.

In this study, the roles of HOXB7 and miR-337 in PDAC were examined by analyzing 44 cases of PDAC patients retrospectively between 2002 and 2006, detecting the expression of HOXB7 mRNA and miR-337, and assessing the relationship between HOXB7 mRNA and miR-337 and survival time.

## Materials and methods

### Clinical sample collection

PDAC samples were obtained from 44 patients diagnosed with PDAC between January 2002 and December 2006 at the First Affiliated Hospital of Zhengzhou University and Wuhan General Hospital of Guangzhou Military. Patients who had PDAC but had received chemoradiotherapy before surgical operation were excluded. Medical records of the 44 patients provided information regarding gender, age, tumor diameter, position, histology, pathological stage, TNM stage, degree of lymph node metastasis and survival time. Of the 44 patients, 28 were male and 16 were female. There were 20 cases with lymph node metastasis and 20 cases without lymph node metastasis. Forty-four paired PDAC and non-malignant adjacent tissues (located more than 5 cm from the tumors) were obtained from PDAC patients who underwent primary surgical resection with informed consent. The diagnosis was established through clinical, biochemical, and radiological findings and supported by pathological examinations. These samples were snap-frozen in liquid nitrogen after resection. This study was approved by the Research Ethics Committee of Zhengzhou University, China, and the samples were obtained with all patients’ informed consent (Table 1).

**Table 1:** Clinicopathologic characteristics of the 44 pancreatic ductal adenocarcinoma cases

**Parameter**	**Category**	**n**
Gender	Male	28
	Female	16
Age (years)	<60	26
	≥60	18
Diameter (cm)	<4	33
	≥4	11
Position	Head	31
	Body and tail	12
Differentiation	Well	9
	Moderate	25
	Poor	10
TNM stage	I or II	20
	III or IV	24
Node status	Negative	24
	Positive	20

### Immunohistochemistry

Polyclonal rabbit anti-HOXB7 (Santa Cruz) was used to detect the HOXB7 protein. Immunostaining was performed using the streptavidin-biotin peroxidase complex method. After visualized with DAB coloration kit (Fuzhou Maixin) and counterstained with hematoxylin. The sections were dehydration, transparency, and mounting. Reactions without primary antibodies served as control reactions.

### Quantitative real-time PCR (qRT-PCR)

Relative levels of miR-337 mRNA in PDAC tissues were determined by quantitative real-time RT-PCR. Total RNA was extracted using an RNA Extraction Kit (Qiagen) and cDNA was extracted using a miScript Reverse Transcription Kit (Qiagen), all according to the manufacturer’s instructions. Relative expression of miR-337 was calculated using the comparative cycle threshold (CT) (2^-△△Ct^) method with U6 snRNA as the endogenous control to normalize the data. For the detection of HOXB7 mRNA, reverse transcription and PCR were performed using ABI TaqMan PCR Master Mix (Applied Biosystems). β-actin was amplified in parallel and served as an internal control. The primers used were as follows: HOXB7, 5′ AGCAGAGGGAC TCGGACTTG 3′ (foward), 5′ CTCGTTTGCGGTCAGTTCCT 3′ (reverse), Probe 5′ FAM-TAACTTCCGGATCTACCCCTGGATGCG-TAMMR 3′; β-actin, 5′ TTCACTTCTTCAGTTCTGCCATCT 3′ (foward), 5′ CCAAGCTTTTCTCAGTCC CATAA 3′ (reverse), Probe 5′ FAM- TCAAAGGGCTCCAGCCTCACTCAGTC -TAMMR 3′. The corresponding CT values were recorded with ABI Prism7500 SDS Software, and then the relative expression level of HOXB7 was calculated according to the formula: 2^-△△Ct^.

### Statistical analysis

Statistical analysis was performed using SPSS17.0 software. Data were expressed as means ± standard deviation (SD). Student’s t test was used to compare of the mean between two samples. The relationships between HOXB7 (or miR-337) expression and clincopathologic characteristics were tested using Chi square test. Survival curves were plotted by Kaplan-Meier method and compared using the log-rank test. *P* values less than 0.05 were considered statistically significant.

## Results

### HOXB7 mRNA was over-expressed in PDAC samples

**Figure 1:**
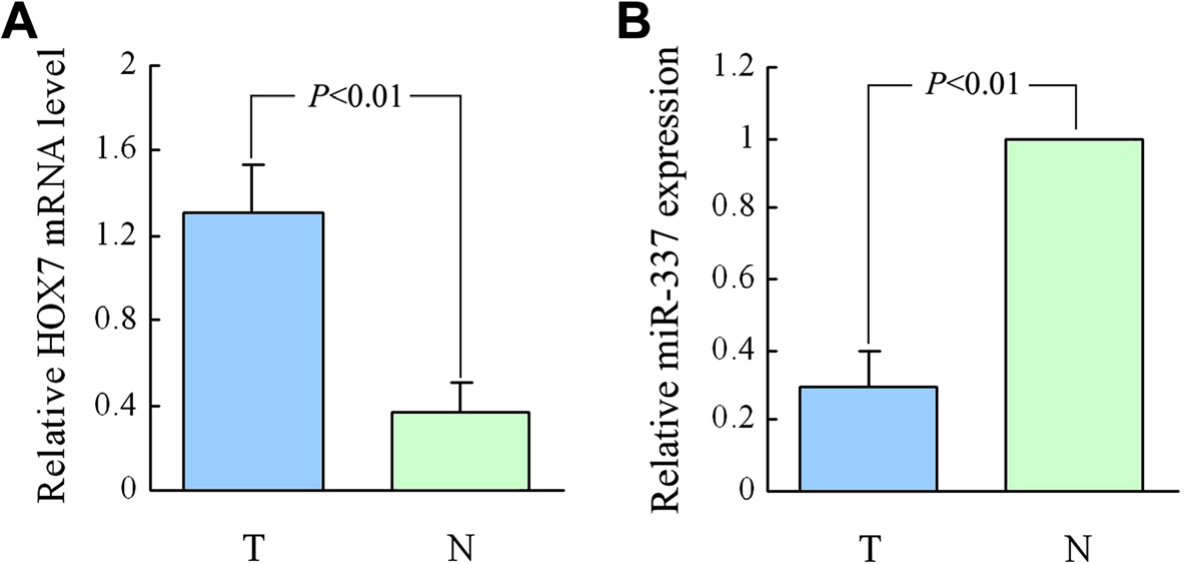
**HOXB7 mRNA and miR-337 expression in 44 human PDAC tissues.** HOXB7 mRNA and miR-337 were detected using qRT-PCR. **A) **Relative level of HOXB7 mRNA in tumor tissues increased compare to non-malignant adjacent tissues (*P*<0.01). **B)** The relative level of miR-337 in tumor tissues was lower than in non-malignant adjacent tissues (*P*<0.01). *T* human PDAC tissues, *N* non-malignant adjacent tissues.

The relative expression level of HOXB7 mRNA was significantly higher in 44 PDAC tissue samples (*P*<0.01, 1.313 ± 0.218) than that in non-malignant adjacent tissues (0.368 ± 0.138) (Figure 1). Statistical differences were found between different diameter (*P* = 0.033), differentiation (*P* = 0.028), TNM stage (*P* = 0.010), and lymph node status (*P* = 0.014). There were no statistically significant differences between patients of different genders (*P* = 0.795), ages (*P* = 0.631), or tumor sites (*P* = 0.690) (Table 2).

**Table 2:** Expression of HOXB7 mRNA, protein, and miR-337 in the pancreatic ductal adenocarcinoma cases

**Parameter**	**HOXB7 protein**			**HOXB7 mRNA**		**miR-337**	
	**Negative (n)**	**Positive (n)**	***P *** **value**	**Expression level**	***P *** **value**	**Expression level**	***P *** **value**
Gender							
Male	10	18	0.764	1.319 ± 0.221	0.795	0.2900 ± 0.09854	0.860
Female	5	11		1.302 ± 0.218		0.2981 ± 0.08573	
Age (years)							
<60	9	17	0.930	1.296 ± 0.229	0.631	0.306 ± 0.105	0.132
≥60	6	12		1.338 ± 0.203		0.274 ± 0.072	
Diameter(cm)							
<4	14	19	0.043*	1.259 ± 0.219	0.033*	0.305 ± 0.097	0.228
≥4	1	10		1.474 ± 0.107		0.257 ± 0.072	
Location							
Head	12	19	0.398	1.308 ± 0.222	0.690	0.304 ± 0.095	0.521
Body or tail	3	9		1.325 ± 0.215		0.263 ± 0.084	
Differentiation							
Well	6	3	0.009**	1.221 ± 0.142	0.028*	0.312 ± 0.082	0.210
Moderate	9	16		1.279 ± 0.243		0.305 ± 0.102	
Poor	0	10		1.481 ± 0.084		0.245 ± 0.066	
TNM stage							
I or II	11	9	0.008**	1.161 ± 0.214	0.010*	0.338 ± 0.109	0.002**
III or IV	4	20		1.440 ± 0.117		0.256 ± 0.057	
Nodal status							
Negative	12	12	0.015*	1.199 ± 0.220	0.014*	0.330 ± 0.105	0.001**
Positive	3	17		1.450 ± 0.111		0.248 ± 0.048	

*P <0.05, **P <0.01.

### HOXB7 protein levels were high in PDAC samples

**Figure 2:**
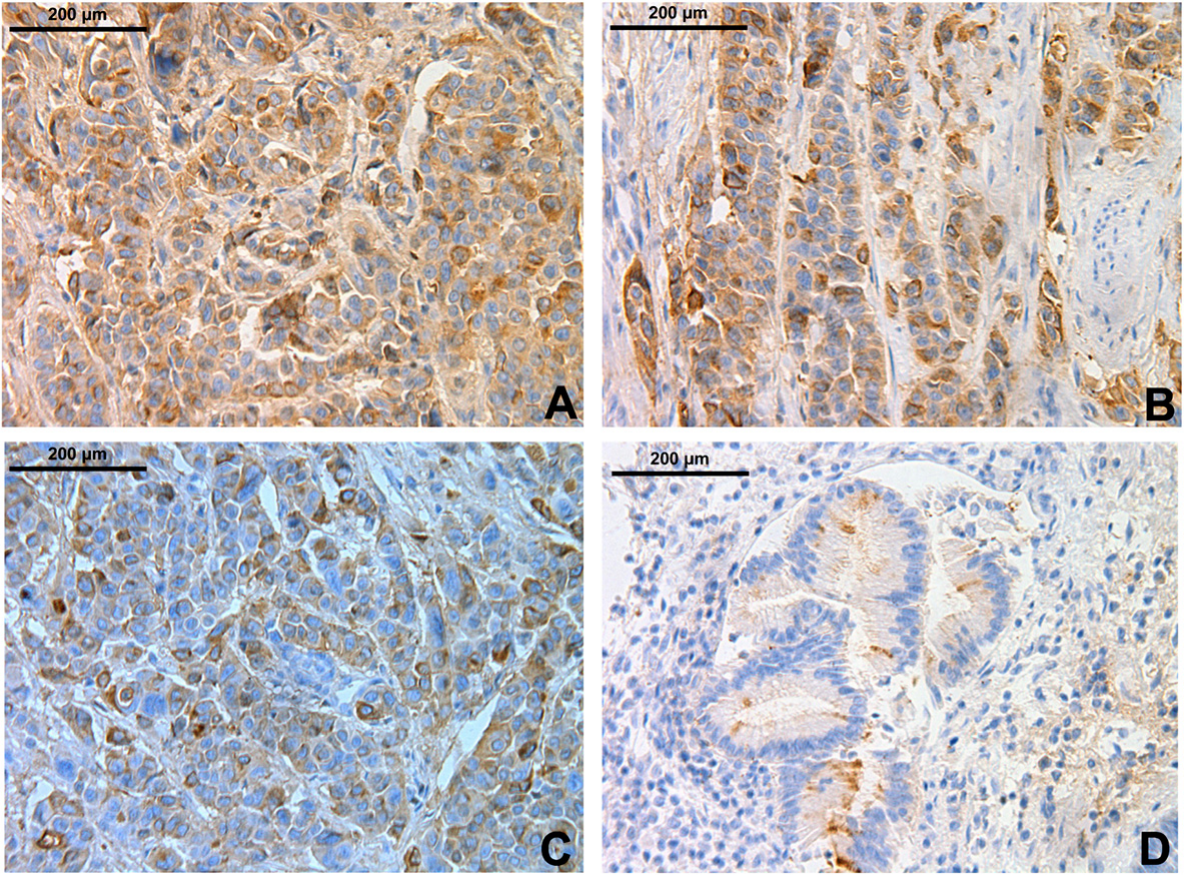
**Immunohistochemical staining of HOXB7 in PDAC tissue sections. A)** and **B)** HOXB7 was strongly positive. **C)** HOXB7 was positive. **D)** HOXB7 was weakly positive.

HOXB7 showed positive immune-reactivity, primarily in the cytoplasm of the cells (Figure 2). The relative amount of positive HOXB7 protein was significantly higher in PDAC tissue samples (*P*<0.01, 65.9%) than in non-malignant adjacent tissues (0%). Elevated expression of HOXB7 protein in the tumor was correlated with different tumors of different diameters (*P* = 0.043), levels of differentiation (*P* = 0.009), TNM stage (*P* = 0.008), and lymph node status (*P* = 0.015). The expression of HOXB7 protein was not associated with genders (*P* = 0.764), age (*P* = 0.930), or tumor site (*P* = 0.398) (Table 2).

### miR-337 levels were low in PDAC samples

The relative expression level of miR-337 was markedly lower in PDAC tissue samples (*P*<0.01) than in corresponding non-malignant adjacent tissues (Figure 1). Low expression of miR-337 was found to be related to TNM stage (*P* = 0.002) and lymph node status (*P* = 0.001). The expression of miR-337 was not found to be related to gender (*P* = 0.860), age (*P* = 0.132), tumor diameter (*P* = 0.228), tumor site (*P* = 0.521) or differentiation (*P* = 0.210) (Table 2).

### Expression of HOXB7 mRNA was negatively correlated to expression of miR-337

Pearson’s product moment correlations were used to analyze the relationship between the level of HOXB7 mRNA and the level of miR-337 in 44 PDAC tissue samples. The level of HOXB7 mRNA in cancer had a negative correlation with the level of miR-337 (*r* = -0.719, *P*<0.01).

### Expression levels of HOXB7 and miR-337 were associated with longer survival in patients with PDAC

**Figure 3:**
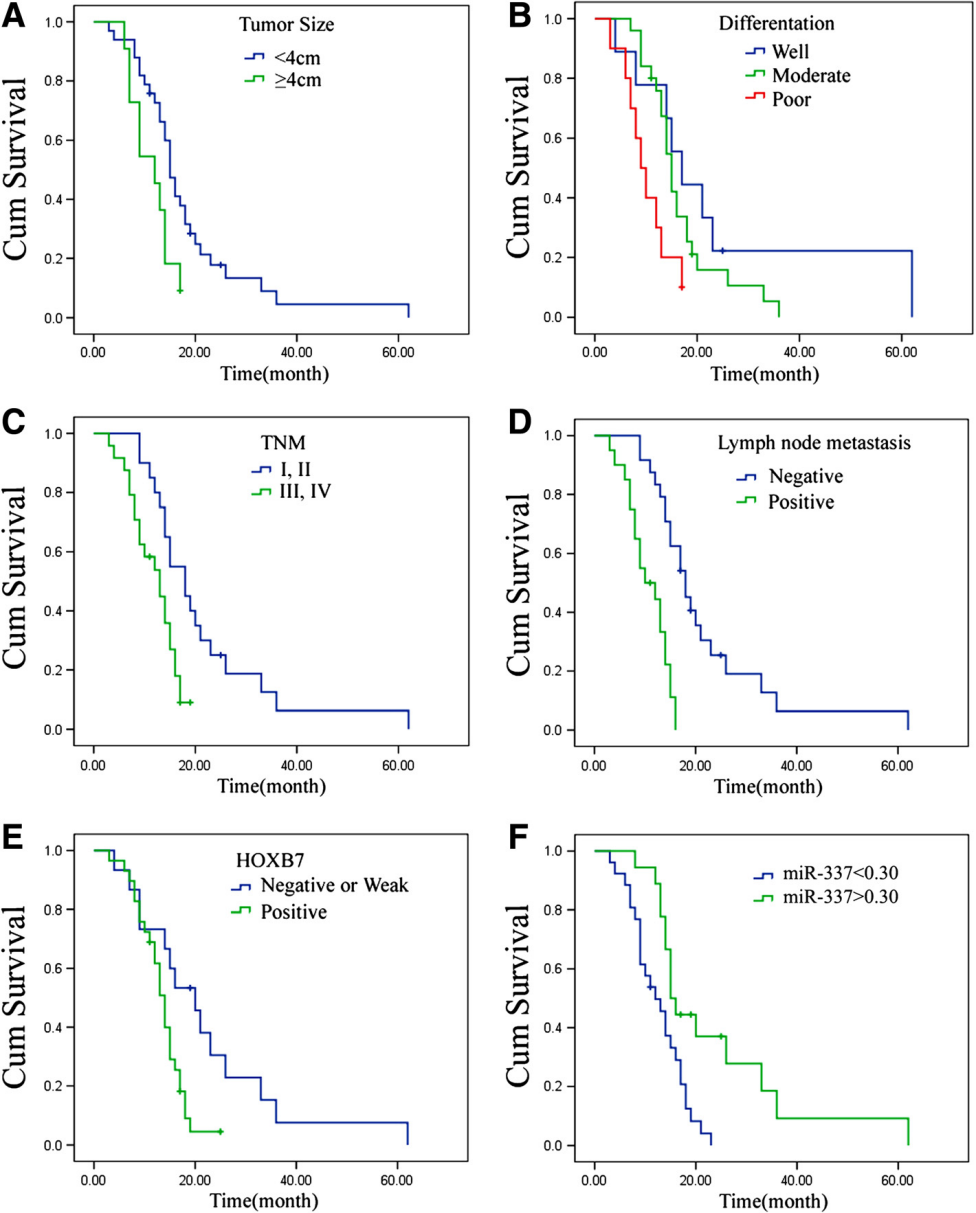
**Kaplan-Meier curves of the clinical outcome in PDAC patients. A)** Survival curves of different tumor size in patients with PDAC (*P*=0.021). **B)** Survival curves of differentiations in patients with PDAC (*P*=0.016). **C)** Survival curves of different TNM stages in patients with PDAC (*P*=0.003). **D)** Survival curves of lymph node metastatic group and non-metastatic group in patients with PDAC (*P*=0.000). **E)** Survival curves of different HOXB7 protein level groups in patients with PDAC (*P*=0.012). **F)** Survival curves of different miR-337 level groups in patients with PDAC (*P*=0.002).

Survival curves were drawn using SPSS 17.0 software using the Kaplan-Meier method. Significant differences in survival curves were observed between patients with <4 cm tumors and patients with ≥4 cm tumors (χ^2^ = 5.306, *P* = 0.021), among groups with good, moderate, and poor differentiation (χ^2^ = 8.322, *P* = 0.016), between groups with TNM stages of I, II and either III, IV (χ^2^ = 8.958, *P* = 0.003), between the lymph node metastatic group and non-metastatic group (χ^2^ = 21.340, *P* = 0.000), among groups in which HOXB7 protein expression was negative (or weak) and positive (χ^2^ = 6.256, *P* = 0.012), between groups with low levels of miR-337 and expression and those in which miR-337 was over-expressed (χ^2^ = 9.181, *P* = 0.002). However no significant differences were observed between male and female patients (χ^2^ = 0.615, *P* = 0.433), between patients <60 years old and ≥60 years old (χ^2^ = 2.857, *P* = 0.091), or between pancreatic head carcinoma and pancreatic body or tail carcinoma (χ^2^ = 0.169, *P* = 0.681) (Figure 3).

## Discussion

Pancreatic cancer has extremely poor prognosis. Many protein, DNA, and RNA-based markers have been shown to be related to the diagnosis and prognosis of pancreatic cancer. MIB-1 (directed against the Ki-67 antigen) helps to determine neuroendocrine pancreatic cancer grade and prognosis [31]. The expression of MUC1 is related to poor prognosis in pancreatobiliary pancreatic head tumors. The expression of MUC5AC is associated with good prognosis and little vascular invasion of PDAC [32].

Previous studies have shown overexpression of HOXB7 to be closely associated with the clinical progression and poor prognosis of patients with breast cancer, oral squamous cell carcinomas, ovarian cancer, lung adenocarcinoma, and esophageal squamous cell cancer [33-36]. The expression of HOXB7 is visibly related to the invasive and aggressive characteristics of human colorectal cancer (high Dukes stage, T stage, distant metastasis-positive tumors, and high proliferation index) and to poor survival of patients [37]. High levels of HOXB7 expression have been detected in both PDAC cell lines and patient samples. HOXB7 knockdown and overexpression in PDAC cell lines caused decreased and increased invasion, respectively [38]. HOXB7 was found to be involved in pancreatic cancer cell proliferation and apoptosis by siRNA assay [39]. High HOXB7 protein expression in the tumor tissues was recently found to be correlated with lymph node metastasis and to be independent predictor of shorter overall survival [38]. This study was performed using immunohistochemical techniques on a large cohort of PDAC.

Using a tri-modal approach, HOXB7 was identified as the target for miR-337. In turn, vascular endothelial growth factor (VEGF) was found to be a downstream transcript regulated by HOXB7 [40]. miR-337 down-regulation was found to promote cellular proliferation, clonogenicity, migration, and invasion and increase tumor cell proliferation and angiogenesis in vivo. It was also found to be closely associated with shorter disease-free survival for cervical cancer. Considering the many reports showing the clinical relevance of expression of HOXB7 and miR-337, it is likely that both HOXB7 and miR-337 are co-regulatory molecules and that they are involved in the carcinogenesis and progression of tumors. The present study confirmed HOXB7 overexpression and miR-337 downregulation in PDAC samples. Significantly higher levels of HOXB7 mRNA and protein were detected in PDAC samples than in non-malignant adjacent tissues. Levels of HOXB7 mRNA and protein expression were positively correlated with diameter, differentiation, TNM stage, and lymph node status, but it was not correlated with gender, age, or position. miR-337 showed markedly less expression in PDAC tissue samples than in non-malignant adjacent tissues. Declines in the expression of miR-337 were found to be related to TNM stage and lymph node status but not to gender, age, diameter, position, or differentiation. The level of HOXB7 mRNA showed a negative correlation with the level of miR-337 in PDAC samples. There were significant differences in survival curves between patients with tumors <4 cm in diameter and patients with tumors ≥4 cm in diameter, among groups of tumors that are well, moderately, and poorly differentiated, between groups with TNM stages I, II and III or IV, between lymph node metastatic group and non-metastatic group, among groups of negative (or weak) and positive for HOXB7 protein expression, between groups with low levels of miR-337 expression and miR-337 over-expression but no significant differences between patients of different sexes, ages, and tumor sites. In conclusion, the present findings not only suggest that the existence of HOXB7 overexpression and miR-337 downregulation in PDAC samples but also that the expression of HOXB7 and miR-337 is correlated with features related to poor prognosis in PDAC patients. Altered expression levels of HOXB7 and miR-337 may be important to invasion and metastasis of PDAC, and HOXB7 and miR-337 may be suitable for use as markers in the assessment and identification of high-risk PDAC patients.

## Abbreviations

PDAC: Pancreatic ductal adenocarcinoma; HOXB7: Homeobox B7.

## Competing interests

The authors declare that they have no competing interests.

## Authors’ contributions

GQZ, RZ and SGZ conceived of the study, participated in its design and coordination, and helped to draft the manuscript. SGZ, YWD, YYW and WQZ collected the samples. SGZ, RZ, YWD, YYW, and WQZ carried out some of the experiments and composed the manuscript. YYW, SGZ, RZ and GQZ performed the statistical analysis. All authors have read and approved the final manuscript.
